# Milk Kefir Beverage Improves Histomorphometry, Reduces Inflammatory Infiltrates and *Desulfovibrio* and Increases *Lactobacillus* in IL‐10^−/−^ Mice

**DOI:** 10.1111/1750-3841.70879

**Published:** 2026-02-10

**Authors:** Iasmim Xisto Campos, Vinicius Fernandes Paris, Mariana de Fátima Albuquerque Pereira, Gabriela de Cássia Ávila Alpino, Andressa Ladeira Bernardes, Larissa Gabriela Morais de Ávila, Marcella Duarte Villas Mishima, Manoela Maciel dos Santos Dias, Soraya Torres Gaze Jangola, Tiago Antonio de Oliveira Mendes, Maria do Carmo Gouveia Peluzio

**Affiliations:** ^1^ Department of Nutrition and Health Federal University of Viçosa—UFV Viçosa Minas Gerais Brazil; ^2^ Department of Nutrition Federal University of Juiz de Fora—UFJF Juiz de Fora Minas Gerais Brazil; ^3^ Interunit Postgraduate Program in Bioinformatics Federal University of Minas Gerais—UFMG Belo Horizonte Minas Gerais Brazil; ^4^ Department of Food Technology Federal University of Viçosa—UFV Viçosa Minas Gerais Brazil; ^5^ Cellular and Molecular Immunology Group René Rachou Institute, Oswaldo Cruz Foundation—FIOCRUZ Belo Horizonte Minas Gerais Brazil; ^6^ Department of Biochemistry and Molecular Biology Federal University of Viçosa—UFV Viçosa Minas Gerais Brazil

**Keywords:** colitis, dairy products, inflammation, intestinal microbiota, probiotics

## Abstract

This study aimed to evaluate the effects of milk kefir on parameters related to inflammatory bowel disease (IBD) in interleukin‐10 knockout (IL‐10^−/−^) mice. Sixteen C57BL/6J IL‐10^−/−^ male mice were divided into two experimental groups. The control group (*n* = 8) received 0.4 mL of whole milk (UHT) and the kefir group (*n* = 8) received 0.4 mL of a fermented beverage made from milk kefir grains (2.4 × 10^8^ colony‐forming units [CFU]), both administered via gavage for 4 weeks. Feces were collected and body weight was measured weekly. At the end of the study, the animals were anesthetized and euthanized, and the small intestine and cecum content were collected for analysis. Proximate composition of kefir and microbiological analysis were conducted. Histomorphometry measurements and quantification of short‐chain fatty acids (SCFAs) were conducted in small intestine. SFCAs were also evaluated in cecum content, and microbiota composition was evaluated in fecal samples. The kefir beverage improved histomorphometry characteristics of the small intestine, increased the number of goblet cells, reduced inflammatory infiltrates, increased *Lactobacillus*, and reduced *Desulfovibrio*, suggesting attenuation of the inflammatory process. Furthermore, the kefir beverage increased the concentration of butyrate, a short‐chain fatty acid with anti‐inflammatory properties, in the small intestine, which may also be associated with reduced inflammation in this group.

## 1 Introduction

Inflammatory bowel disease (IBD) is a chronic disorder that can affect any part of the gastrointestinal tract (Chou et al. 2019; Sairenji et al. 2017; Ng et al. 2017). The term comprises the disorders of Crohn's disease (CD) and ulcerative colitis (UC), which differ from an anatomical and histological perspective (Hadji and Bouchemal 2022). The pathogenesis is multifactorial, with an impact from the association between genetics and environmental factors and intestinal microbiota (Dai et al. 2023; Ananthakrishnan 2015). Due to the different related events, the development of efficient therapies to combat IBD is a challenge (Venegas et al. 2019).

The intestinal microbiota is directly related to the development of IBD, given that the same factors influencing microbiota composition can also have an impact on IBD, such as breastfeeding, antibiotics, and diet (Pittayanon et al. 2020; Qiu et al. 2021). Therefore, alterations in the composition or functionality of the microbiota, such as a reduction in alpha bacterial diversity and a decrease in the production of short‐chain fatty acids (SCFAs), can influence the progression of the disease (Qiu et al. 2021; Kostic et al. 2014; Russo et al. 2019).

Regarding treatment strategies in IBD, the use of probiotics stands out, and one of the potential mechanisms they are involved in is their immunomodulatory effect and anti‐inflammatory properties (Lê et al. 2022; Selvamani et al. 2022). In this context, kefir is a fermented beverage, which is obtained through fermentation by a protocooperation of bacteria and yeasts, known as “kefir grains,” that settle in a substrate matrix with milk or water (Azizi et al. 2021; de Souza et al. 2024). As the beverage obtained by kefir grains fermentation exhibits probiotic microbial species, recent studies in the literature place the application of kefir with the potential to obtain probiotic foods with probiotic effects, along with the production of bioactive compounds such as bioactive peptides, amino acids, SCFA, enzymes, and bacteriocins, adding beneficial properties to the beverage (de Souza et al. 2024; Pihurov et al. 2021). Experimental models of intestinal inflammation have been used in studies about the pathophysiology of diseases and the development of new therapeutics. Interleukin‐10 knockout mice (IL‐10^−/−^) develop spontaneous chronic inflammation (Mizoguchi 2012), affecting the entire gastrointestinal tract but predominantly the colon region, resembling IBD in humans from a histopathological perspective (Elson et al. 1995; Davidson et al. 1996).

Therefore, the present study aimed to evaluate the effects of milk kefir beverage consumption on parameters related to IBD in an experimental model of (IL‐10^−/−^) mice.

## 2 Materials and Methods

### 2.1 Preparation of the Kefir Beverage

The kefir grains were obtained from families who traditionally consume them in the city of Viçosa, Minas Gerais, Brazil. The production of the fermented beverage was carried out at the Laboratory of Nutritional Biochemistry, Department of Nutrition and Health (DNS) at Federal University of Viçosa (UFV).

**FIGURE 1:**
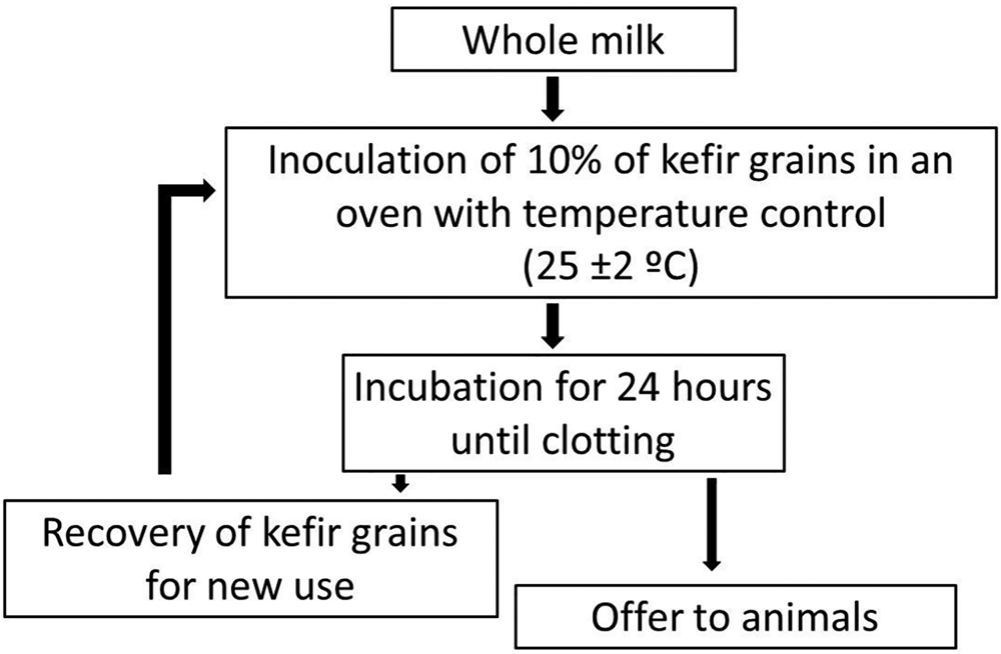
Milk kefir production flowchart.

To produce fermented kefir beverage, the grains were separated into portions and 10% of the kefir grains were inoculated into commercially obtained whole cow's milk (UHT) from a single brand and batch. After inoculation, the milk containing the kefir grains was kept at a controlled temperature of 25°C ± 2°C for 24 h, without agitation (D. D. Rosa et al. 2016). The grains were separated from the fermented milk (kefir), washed with distilled water, and reused for a new production successively. The product obtained from kefir fermentation was offered to the animals during the intervention period (Figure 1).

The same fermentation protocol, using the same kefir grains, was performed daily during all the experimental design period, ensuring that the mice consumed a similar product (10^8^ colony‐forming units [CFU]/mL for lactic acid bacteria (LAB) and 10^4^ CFU/mL for yeasts).

### 2.2 Proximate Composition of Kefir and pH Evaluation

The fermented kefir beverage was freeze‐dried. The difference in weight between the liquid and dried products was used to calculate moisture content. The powdered product was used for the assessment of proximate composition (protein, lipids, ashes, and carbohydrates). All the analyses were conducted following methods described by AOAC (Association of Official Analytical Chemistry 2005) and performed in triplicate. The quantity of carbohydrates was inferred by difference. The pH of the fermented kefir beverage was determined using a pH meter (Instrutherm, model PH‐1900, Brazil) (Instituto Adolfo Lutz 1985).

### 2.3 Microbiological Analysis

To assess the viability of kefir as a probiotic beverage, it was necessary to evaluate the quantity of LAB in the beverage. Using the microdrop technique, 20 µL of serial dilutions of kefir were inoculated on the surface of each plate containing Man, Rogosa, and Sharpe agar medium (MRS). The plates were then incubated at 37°C for 48 h in an incubator for CFU counting. Yeast counting was also performed using the surface plating method with serial dilutions. On the surface of each plate containing potato dextrose agar (PDA) acidified with a 10% tartaric acid solution, 100 µL of decimal dilutions of kefir were inoculated. The plates were then incubated at 25°C in an incubator for 5 days. Using the formula: (final mean CFU count × dilution factor)/volume used for plating, the quantity of CFU/mL of kefir was determined (Ministério Da Agricultura, Pecuária e Abastecimento 2003).

### 2.4 Experimental Design

Sixteen C57BL/6J IL‐10^−/−^ male mice at 8 weeks old, weighing approximately 24 g, were obtained from the Central Animal Facility at the Center for Biological and Health Sciences (CCB) at UFV, with approval from the Ethics Committee for Animal Use (CEUA/UFV) under protocol number 52/2019. The sample size was calculated according to Mera et al. (1998), and no animals were excluded. A confidence level of 99% was used (*α* < 0.01), obtaining a value from the distribution table *t* (two‐tailed) = *t_ɑ_*/2 = 2,5 with a statistical power of 95% and 20% of losses.

**FIGURE 2:**
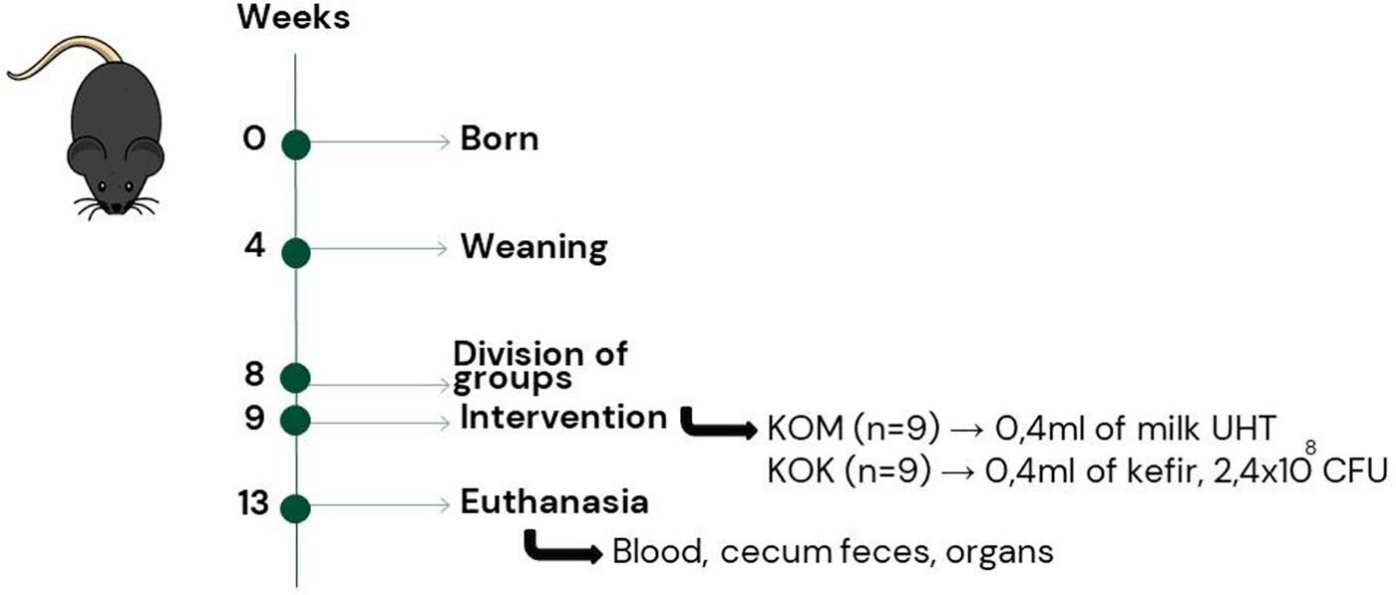
Experimental design. CFU, colony‐forming units; KOK, kefir group; KOM, control group.

The animals were housed in 4 collective cages, 2 for each group, under a 12‐h light/dark cycle, at 23 ± 2°C and provided with water and commercial feed ad libitum throughout the experiment. The animals were randomized based on weight into two experimental groups: control group (KOM, *n* = 8) and the kefir group (KOK, *n* = 8). After a week of acclimatization, the control group (KOM) received 0.4 mL of whole cow's milk (UHT) and the kefir group (KOK) received 0.4 mL of fermented kefir beverage (2.4 × 10^8^ CFU) both by orogastric gavage, daily, for 4 weeks (Du et al. 2022) (Figure 2). The animals were individually weighed weekly on a digital scale. At the same time, excreted feces were collected and promptly placed in the ultrafreezer at −80°C.

At the end of the experimental period, the animals were weighed, anesthetized with 3% isoflurane (Isoflorine, Cristalia), and euthanized by exsanguination. The small intestine was removed, flushed with physiological saline solution, weighed, and stored at −80°C until the analysis or fixed in Carson's formalin (Carson et al. 1973) for histological analysis. The content from the cecum was also removed and stored at −80°C until the analysis.

### 2.5 Histological Analyses in the Small Intestine

The tissue was embedded in the paraffin. Subsequently, 5 µm‐thick sections were obtained using a rotary microtome (Olympus America Inc., CUT 4055). These sections were then stained with hematoxylin and eosin (H&E). Images of the histological sections were captured with a 10x objective directly on the LEICA DM750 light microscope (Leica, Switzerland) using a LEICA 170HD video camera (Leica, Switzerland). Analyses and measurements were performed using the Image Pro‐Plus version 4.5 software (Media Cybernetics).

The histopathological score in small intestine was composed of the average of three different parameters: crypt damage (0: none; 1: 1/3 damaged; 2: 2/3 damaged; 3: only the superficial epithelium intact; 4: epithelium lost); inflammation severity (0: none; 1: mild; 2: moderate; 3: severe); and lesion depth (0: none; 1: mucosa; 2: submucosa; 3: transmural). A score was assigned for each parameter, and the overall score was obtained by summing the points for each parameter (Cruz et al. 2020).

Histomorphometry measurements were conducted using Image Pro‐Plus version 4.5 (Media Cybernetics), following the methodology proposed by D. D. Rosa et al. (2012). Goblet cells were manually counted in 70 villi for each experimental group.

### 2.6 Quantification of SCFAs in the Small Intestine and in Cecum Content

SCFAs were extracted following the methodology proposed by Siegfried et al. (1984), and the concentration was quantified by HPLC on an UltiMate 3000 chromatograph (Dionex, Germany). Twenty microliters of the sample homogenate were injected and passed through a Rezex ion‐exchange column (00H‐0138‐K0 300 × 7.8 mm, Phenomenex) with 5 mM sulfuric acid as the eluent at 0.7 mL/min, at 40°C. The detector used was a refractive index detector at 40°C (RI‐101, Shodex). The concentration of fatty acids was determined based on the standard curve of acetic, propionic, and butyric acids, and the result expressed in µmol of fatty acid/g of tissue or µmol of fatty acid/g of cecum content.

### 2.7 Analysis of Intestinal Microbiota Composition

For sequencing, fecal samples from the first and last week of the intervention were used. Fecal DNA extraction was performed as described by Zhang et al. (2006), using 30 ± 2 mg of feces. Collected feces from the animals were distributed into three pools per group. The concentration and integrity of bacterial DNA were assessed using NanoDrop, with the calculation of the 260/230 and 260/280 ratios, absence of degradation smear in 0.8% agarose gel electrophoresis, and positive PCR amplification using 337F and 518R 16S rRNA primers for the V3 hypervariable region to detect 16S rRNA (Park et al. 2021). The samples were then sent to the company Neoprospecta (Florianópolis, Santa Catarina, Brazil) for sequencing on Illumina HiSeq 2500 platform (Caporaso et al. 2012).

The sequence quality was assessed using the FastQC package to remove low‐quality sequences, those with a Phred quality score less than 30, using the Trimmomatic v0.36 program. High‐quality reads were then processed using the DADA2 package version 1.8 implemented in R platform version 3.6.1. Chimeric sequences were identified and removed. The representative OTU sequences were taxonomically classified (phylum, family, and genus) using the Silva 16S rRNA database release 138 (Quast et al. 2013).

### 2.8 Statistical Analyses

The normality of variables was determined using the Shapiro–Wilk test. For comparison between experimental groups with parametric distribution, Student's *t*‐test was employed. Nonparametric tests were used for values that did not exhibit normal distribution. Abundance profiles of the top 50 bacterial taxa were calculated at taxonomic levels from phylum to species, presented as percentage abundance, and compared using either a paired Student's *t*‐test or Wilcoxon test for dependent samples and Student's *t*‐test or Mann–Whitney test for independent samples.

Results were expressed as mean ± standard deviation. Statistical analyses were conducted using the Statistical Package for the Social Sciences (SPSS) version 28.0 for Windows Evaluation Version, assuming a *p* < 0.05. Microbiota analyses were assessed using GraphPad Prism 8 software.

## 3 Results and Discussion

The proximate composition was determined only for the kefir beverage to characterize the fermented product administered to the animals. The UHT milk used as control is a commercially regulated product, subject to standardized quality control and nutritional labeling requirements. As the main objective of this study was to evaluate the biological effects of kefir consumption, the manufacturer's information was considered sufficient. A moisture content was observed as 89%. In terms of macronutrients, 4.50 g of carbohydrates, 3.20 g of protein, and 2.60 g of lipids were found in 100 g of fermented kefir beverage. The ash content was 0.70/100 g, and the total caloric value provided by the beverage was 53.80 kcal per 100 g. Additionally, the pH of the beverage was 4.03. Moreover, the composition of the milk used, according to the manufacturer, was 4.5% carbohydrates, 3% protein, and 3% lipids, providing a total of 57 kcal per 100 g. After plating in serial dilutions, a count of 10^8^ CFU/g of total LAB and 10^6^ CFU/g of total yeast was obtained.

Body weight differed between groups only at specific time points. A significant difference was observed in Week 2, when the KOM group showed higher body weight compared to the KOK group (Table 1). In Week 10, the KOM group exhibited weight gain, whereas the KOK group showed weight loss (*p* = 0.0002). No significant differences were detected between groups in subsequent weeks. At the end of the experiment, all animals in both groups lost body weight, suggesting the development of spontaneous IBD.

**TABLE 1:** Weekly body weight and evolution of weight in interleukin (IL)‐10 knockout mice treated with milk kefir.

Week	Mean of body weight (g)		*p*	Evolution of body weight (g)		*p*
	KOM	KOK		KOM	KOK	
**8**	24.77 ± 0.74	23.67 ± 1.28	0.0538			
**9**	25.65 ± 0.89	24.67 ± 1.32	0.1050			
**10**	26.60 ± 1.08	24.40 ± 1.40	0.0034*	0.96 ± 0.42	−0.27 ± 0.39	0.0002*
**11**	25.96 ± 2.05	24.84 ± 1.33	0.2233	−0.65 ± 1.08	0.46 ± 0.59	0.0232*
**12**	26.09 ± 1.45	25.04 ± 1.18	0.1459	0.33 ± 0.87	0.18 ± 0.53	0.6859
**13**	22.75 ± 1.37	22.28 ± 1.49	0.5200	−3.50 ± 0.44	−2.76 ± 1.34	0.1867

*Note*: Values are expressed as means ± SD, *n* = 3 animals/group. * Indicates differences between groups according to Student's *t*‐test.

Abbreviations: KOK, kefir group; KOM, control group.

**FIGURE 3:**
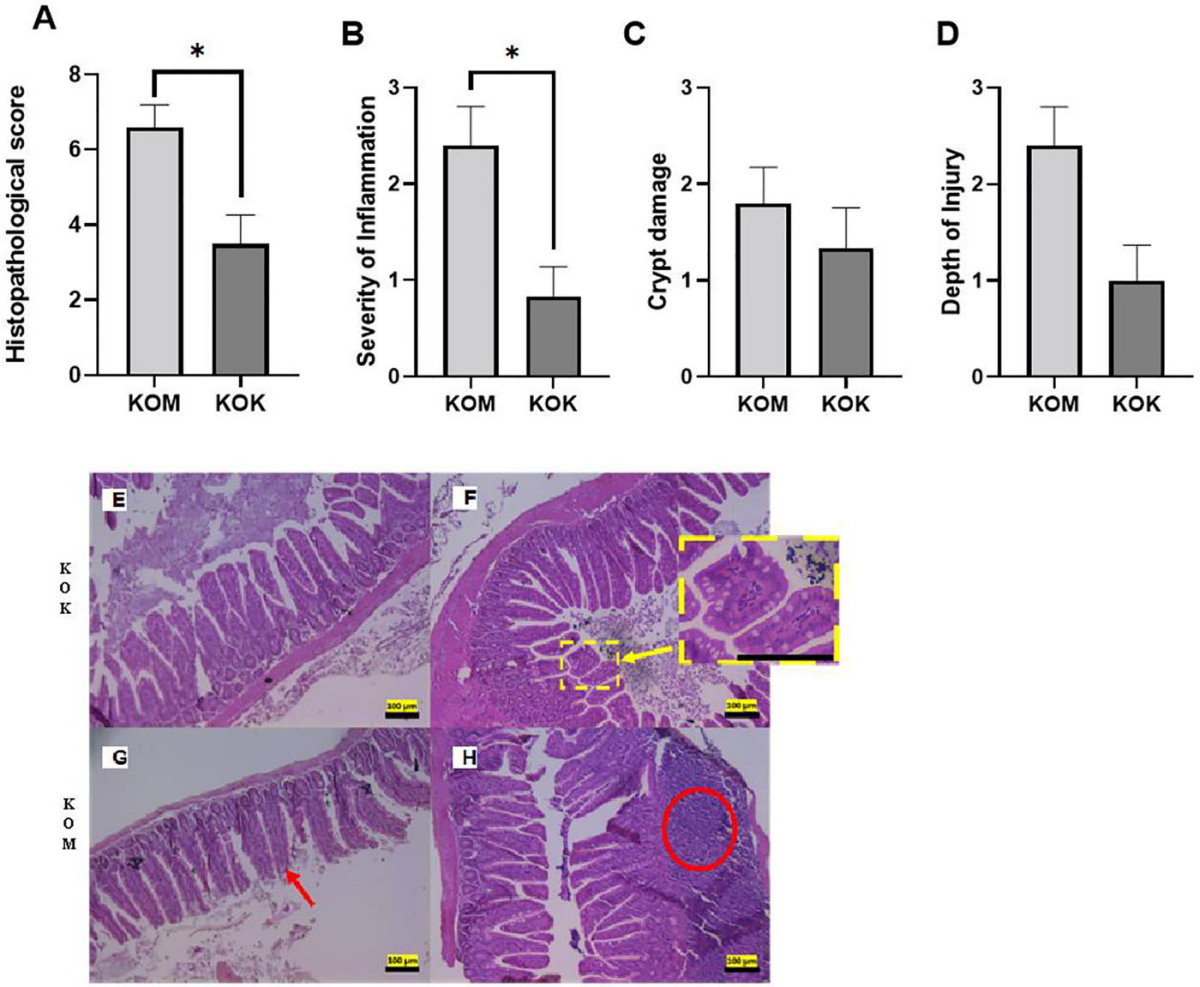
Histopathological score of the use of kefir on the small intestine of IL10^−/−^ mice (eosin–hematoxylin stain). (A) Total histopathological score; (B) severity of inflammation; (C) damage to crypts and villi; (D) depth of lesion. Values expressed as mean ± standard deviation. * Indicates *p* < 0.05 demonstrating statistical difference by Mann–Whitney or unpaired *t*‐test. Photomicrograph of the small intestine of C57BL/6J IL10^−/−^ mice stained with HE. (E) Preserved villi and crypts; (F) evident and enlarged goblet cells; (G) damaged villi and reduction of goblet cells; (H) presence of inflammatory infiltrate. Yellow arrow: goblet cells. Red arrow: flattening and damage to the villi. Red circle: inflammatory infiltrate. Objective 10×. KOK, kefir group; KOM, control group.

**FIGURE 4:**
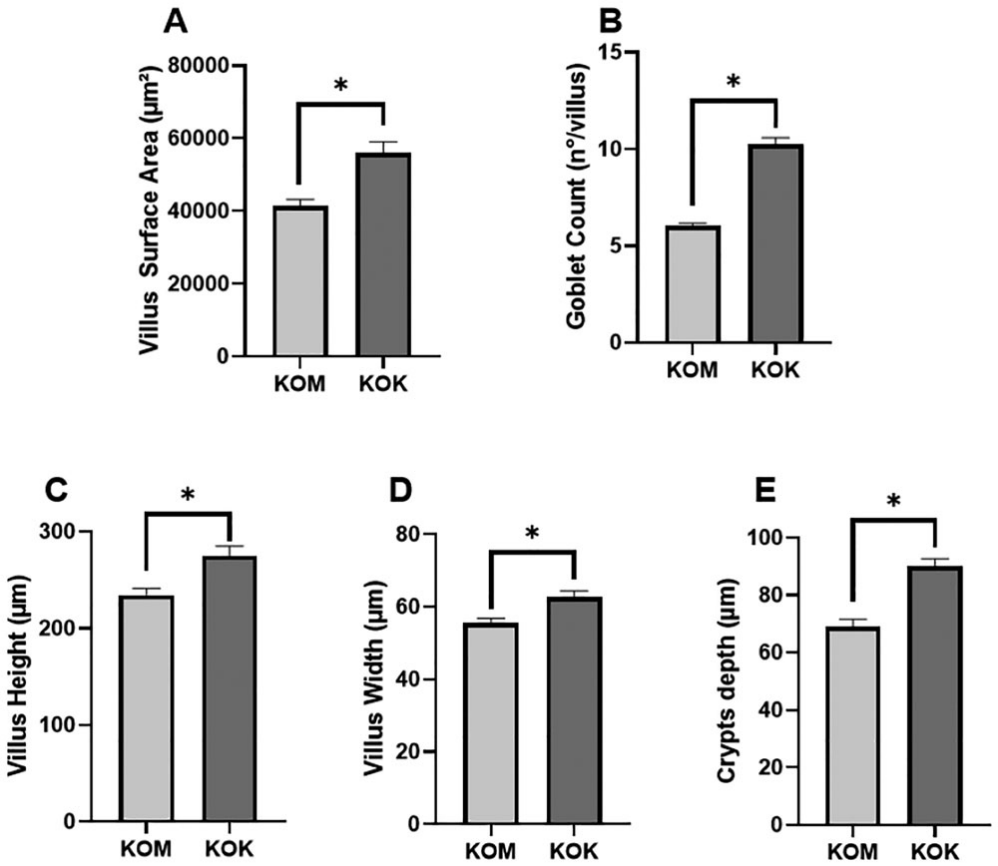
Evaluation of the use of kefir on the histomorphometric variables of the small intestine of IL10^−/−^ mice (eosin–hematoxylin stain). Evaluation of kefir use on histomorphometric variables of the small intestine of IL10^−/−^ mice. (A) Villus surface area; (B) goblet cell count; (C) villus height; (D) villus width; (E) crypt depth. Values expressed as mean ± standard deviation. * Indicates statistical difference *p* < 0.05 by Mann–Whitney. KOK, kefir group; KOM, control group.

In the assessment of histopathological scores for the small intestine (Figure 3A) and the severity of inflammation parameter (Figure 3B), the group fed with kefir demonstrated a reduction in both parameters. Additionally, it was observed that kefir treatment preserved the physiological tissue appearance (Figure 3E,F), maintaining homogeneous villi and reducing focal inflammatory infiltrates. In contrast, the control group exhibited tissue damage and large foci of inflammatory infiltrates between the intestinal epithelium and lamina propria (Figure 3G,H). There was no difference in crypt damage and depth injury (Figure 3C,D). An increase in villus surface area, height and width of villi, and crypt depth was observed (Figure 4A,C–E) in the group KOK. The control group demonstrated a reduction in the number of goblet cells per villus compared to the kefir group (Figure 4B).

**FIGURE 5:**
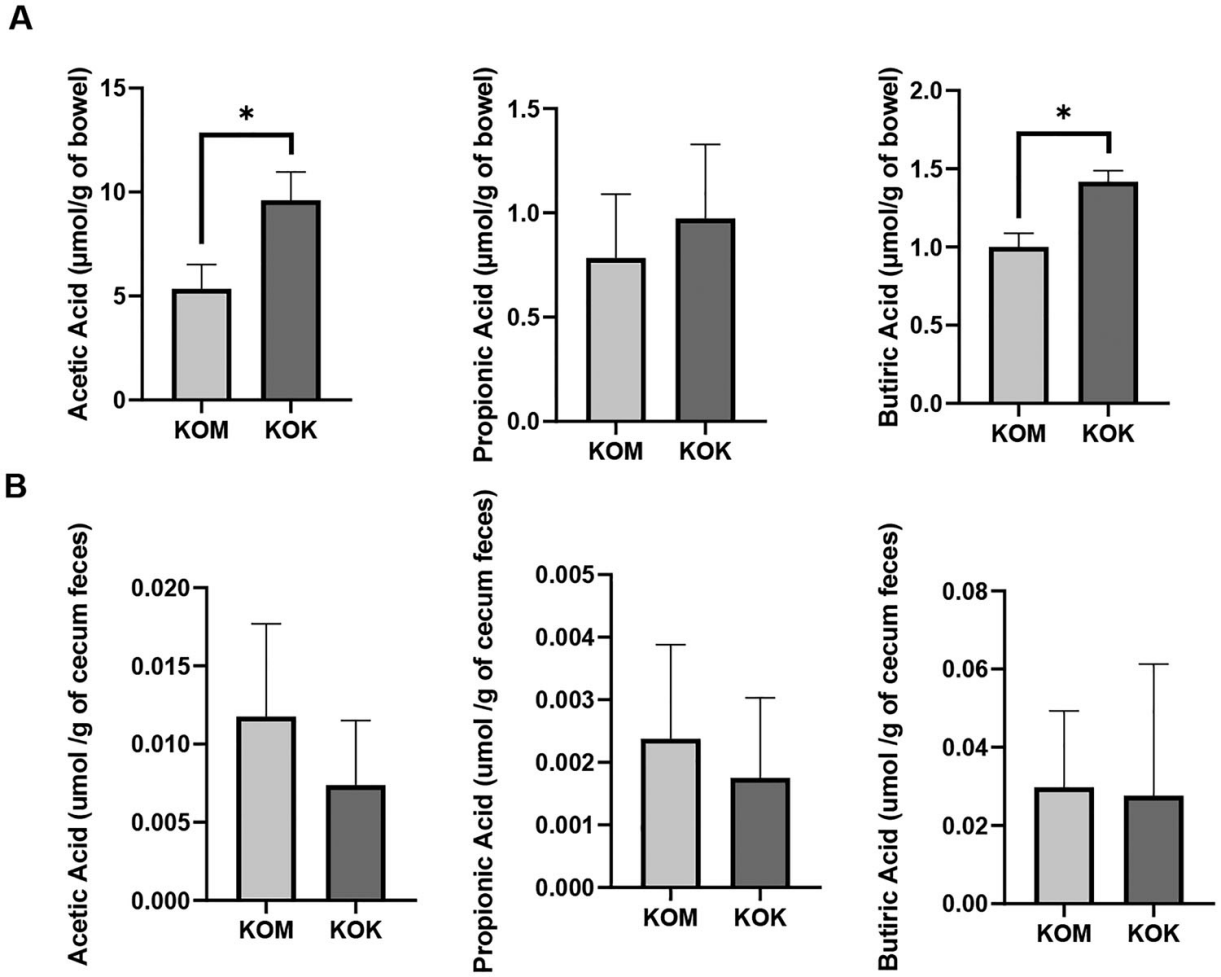
Short‐chain fatty acid (SCFA) concentrations in the small intestine (A) and cecum feces (B) of IL10^−/−^ mice after kefir consumption. Values expressed as mean ± standard deviation. * Indicates statistical difference *p* < 0.05 by Mann–Whitney. KOK, kefir group; KOM, control group.

Kefir treatment increased the concentration of SCFAs in the small intestine, with an increase in acetic and butyric acid concentrations compared to the control group (Figure 5A). The functionality of intestinal bacteria was assessed through the concentration of SCFAs (acetate, butyrate, and propionate) in cecal content and no differences were found in acetate, propionate, and butyrate concentrations between the experimental groups (*p* = 0.11, 0.39, 0.39) (Figure 5B).

**FIGURE 6:**
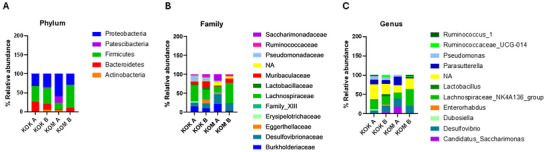
Taxonomic analyses of fecal microbiota of IL10^−/−^ mice at the phylum, family, and genus levels. Taxonomic analyses of fecal microbiota at the phylum, family, and genus levels. (A) phylum relative abundance; (B) family relative abundance; (C) genus relative abundance; KOK A: kefir group in first week of intervention; KOK B: kefir group in fourth week of intervention; KOM A: control group in first week of intervention; KOM B: control group in fourth week of intervention.

The results of the metataxonomic analysis were assessed intragroup (A), meaning a comparison between the first and last week of intervention, and intergroup (B), where the results of the last week of the experiment were compared between experimental groups. The phyla Bacteroidetes, Firmicutes, and Proteobacteria were present in both groups at the beginning and at the end of the experimental period (Figure 6A). The kefir intervention led to a reduction in the Desulfovibrionaceae family compared to the control group, and the Lactobacillaceae family was identified only in the KOK group during the first week of intervention and persisted until the end of the experimental period (Figure 6B). Regarding the genera, an increase in *Lactobacillus* was observed after kefir intervention (Figure 6C). *Desulfovibrio*, the only genus of the Desulfovibrionaceae family identified, was more abundant in the KOM group compared to the KOK group (Figure 6C).

Alterations in the composition and functionality of the microbiota are associated with IBD, and the use of probiotics has been studied as a potential treatment. Kefir is a fermented beverage that contains probiotic microbial species which add beneficial properties to the beverage. Here, we aimed to evaluate the effects of milk kefir beverage consumption on parameters related to IBD in IL‐10^−/−^ mice. Our results demonstrated that, in the small intestine of IL‐10^−/−^ mice, kefir improved histomorphometric characteristics, maintaining normal tissue physiological appearance with homogeneous villi and increased concentrations of acetate and butyrate. An increase in Lactobacillaceae family and *Lactobacillus* genus was observed, whereas the Desulfovibrionaceae family and *Desulfovibrio* genus decreased, also suggesting attenuation of the inflammatory process.

In our study, we used whole milk kefir due to its better‐established scientific knowledge. The proximate composition of kefir demonstrated an expected moisture content for a beverage (89%). A similar value, 90% moisture, was found in another study from our group (D. D. Rosa et al. 2017) and 87.5% in a review conducted by Otles and Çağındı (2003). Regarding the protein composition, the value found in our beverage (3.20/100 g) is higher than the minimum requirement specified in the legislation for fermented milks in Brazil, which considers a minimum protein content of 2.90/100 g of product (Ministério Da Agricultura, Pecuária e Abastecimento 2007). Similar values, ranging from 3.16 to 3.18 g of protein, were reported (E. R. Farnworth 2008). Considering the use of whole milk as the substrate for kefir, the lipid found is below the recommended range for fermented milks, which is between 3.0 and 5.0 g of lipid per 100 g of product (Ministério Da Agricultura, Pecuária e Abastecimento 2007). According to the same regulation, the fermentation product would be classified as partially skimmed, with a fat content between 0.6% and 2.9%. The reduction in lipid content may result from the action of some yeast species with lipolytic activity present in kefir (Vujičić et al. 1992; Lopitz‐Otsoa et al. 2006). The average pH found was 4.03, a value that classifies it as acidic (below 7.0). This characteristic is achieved through the fermentation process of milk, which induces the production of lactic acid and contributes to the preservation of the obtained product (Leite et al. 2013).

The proximal composition of kefir undergoes variation based on the type of milk, the fermentation process, climatic conditions, and grain management (Sarkar 2008). Its microbiological composition can vary depending on the origin of the grains, the type of substrate used for its development, and its cultivation method (Prado et al. 2015). Here, our microbiological evaluation of kefir demonstrated a count of 10^8^ CFU/g of total LAB and 10^6^ CFU/g of total yeast was obtained. This result suggests that the analyzed kefir possesses possible probiotic properties, as it aligns with the characteristics established by the legislation in Brazil (Ministry of Agriculture, Livestock and Food Supply) in Ministério Da Agricultura, Pecuária e Abastecimento (2007).

In the present study, the body weight loss in the KOK group may be related to inflammation generated. This weight loss is expected due to the degree of inflammation they may reach. In the study conducted by Chen et al. (2017), probiotic treatment was able to improve body weight loss in IL‐10^−/−^ animals compared to the untreated control group. Besides that, in IBD, histological changes in the small intestine, such as partial villous atrophy, crypt/villus ratio alterations, and infiltration of inflammatory cells, can be observed (Mourad et al. 2017). According to our results, kefir had a positive effect on maintaining the tissue characteristics of the small intestine, demonstrating a positive effect. The structure of the villi can increase the surface area, and increasing the height of the villi leads to greater efficiency in the process of digestion and absorption of nutrients. The surface area of the villi and the depth of the crypts are indicators of intestinal development and functional status (Hou and Tako 2018; Núñez‐Gómez et al. 2023; Mishima et al. 2023).

Goblet cells secrete mucus; the mucus layer functions as a crucial barrier and that is responsible for covering the intestinal epithelium and providing the first line of defense against injuries caused by ingested food, microbes, or microbial products. However, the process of infection or chronic inflammation can lead to depletion of goblet cells and changes in the mucosal layers (Liévin‐Le Moal and Servin 2006; Dharmani et al. 2009; Kim and Ho 2010; Pelaseyed et al. 2014). The disruption of this barrier may constitute one of the possible causes of IBD (van der Post et al. 2019; Naama et al. 2023).

The production of SCFA benefits the host as regulatory molecules with various physiological effects, including energy homeostasis, lipid and carbohydrate metabolism, and suppression of inflammatory signals (Hee and Wells 2021; Fu et al. 2023). Furthermore, butyrate may protect against colitis by regulating the production of Treg cells and enhancing the antibacterial activity of macrophages (Haneishi et al. 2023). Therefore, a higher concentration of butyrate could attenuate inflammation in the small intestine. Besides that, it improves the integrity of intestinal epithelial cells, promoting tight junctions and cell proliferation, inhibits the secretion of pro‐inflammatory cytokines while increasing the secretion of anti‐inflammatory cytokines and increases mucin production by goblet cells (Siddiqui and Cresci 2021), which is in accordance with our results. Here, we found higher number of goblet cells and higher concentration of butyrate in small intestine, but we did not find differences in SCFA concentrations in cecal content.

Dysbiosis can be assessed through the variations in Firmicutes to Bacteroidetes ratio (F:B ratio), which is used as a predisposing factor for diseases (Jandhyala et al. 2015). In the present study, the control group demonstrated a decrease in the F:B ratio after 4 weeks of intervention, whereas the kefir intervention, after 4 weeks, increased the F:B ratio, suggesting an imbalance in the microbiota of the control group. Usually, a decreased F:B ratio and a decrease in the abundance of Firmicutes are observed during the active phase of the disease in individuals with IBD (Stojanov et al. 2020; Xue et al. 2022). According to Qu et al. (2021), animals induced with dextran sulfate sodium (DSS) for IBD exhibited a reduction in the abundance of both Firmicutes and Bacteroidetes. Restoring the F: B ratio with proper probiotics can help or support the immune system (Stojanov et al. 2020).

Among the predominant probiotic strains in kefir are bacteria such as *Lactobacillus*, *Lacticaseibacillus*, and *Lactiplantibacillus*, and the yeasts *Saccharomyces*, *Candida kefyr*, and *Kluyveromyces*. Thus, the presence of probiotic species makes the beverage a potential probiotic food matrix (de Souza et al. 2024). In our study, the kefir intervention reduced the Desulfovibrionaceae family, which produces hydrogen sulfide that can compromise the intestinal mucosal barrier and induce IBD (Zhai et al. 2019) and it is more prevalent in individuals with IBD compared to healthy (Nagao‐Kitamoto and Kamada 2017). The Lactobacillaceae family was identified only in the KOK group during the first week of intervention and persisted until the end of the experimental period, and the *Lactobacillus* genera were increased after kefir intervention. Several species of *Lactobacillus* have been found in kefir samples (D. D. Rosa et al. 2017) and it is one of the most found microorganisms in it (Slattery et al. 2019). Several species of *Lactobacillus* are used for IBD treatment, including *L. reuteri*, *L. plantarum*, *L. fermentum*, and *L. casei*. Except for *L. fermentum*, all *Lactobacillus* species have been found or are related to kefir consumption in various outcomes, such as weight loss, reduction of pro‐inflammatory cytokines, changes in cholesterol profile, and reduction of oxidative stress (Peluzio et al. 2021).

*Desulfovibrio* was more abundant in the KOM group compared to the KOK group. This genus has been found in biopsies of patients with UC and it is associated with leaky gut, a compromise in the integrity of the intestinal barrier (Singh et al. 2022). The increase in *Desulfovibrio*, the most prevalent sulfate‐reducing bacteria, is linked to IBD and pouchitis, an inflammation that occurs in the lining of a pouch created during surgery to treat UC or other diseases (Singh et al. 2022). Sulfate‐reducing anaerobic bacteria utilize organic compounds as a source of energy and carbon through a process known as dissimilatory sulfate reduction. This process produces toxic hydrogen sulfide (H_2_S), which, at high concentrations, can induce inflammation of the intestinal epithelium and consequently contribute to the development of colitis (Kushkevych et al. 2020). *Desulfovibrio* bacteria genus utilizes sulfate as a terminal electron acceptor and organic compounds in its metabolism. Therefore, kefir treatment could attenuate the inflammatory process by reducing the abundance of this genus.

Although we identified changes in the composition of the microbiota, we did not observe any alterations in the functionality of SCFA production (concentration of SCFA in the cecal content). This suggests the possibility that altered bacteria may not be responsible for the synthesis of these acids. We can hypothesize that the kefir administered to IL‐10 knockout mice may have influenced species not specialized in SCFA production, or that the duration of intervention and the quantity employed were not sufficient to effectively modulate this specific function.

## 4 Conclusion

In conclusion, the present study demonstrates that the consumption of a whole milk kefir beverage exerted beneficial effects on intestinal health and inflammatory parameters in IL‐10^−/−^ mice, a well‐established model of IBD. The kefir showed an adequate nutritional and microbiological profile in addition to relevant biological effects associated with gut integrity, such as preservation of physiological architecture of the small intestine, improving histomorphometric parameters, while reducing inflammatory infiltrates.

The modulation of the intestinal microbiota further supports the protective effects of kefir. The increase in Lactobacillaceae and *Lactobacillus*, alongside the reduction of Desulfovibrionaceae and *Desulfovibrio*, indicates a shift toward a more favorable microbial profile. This microbial modulation is especially relevant in the context of IBD, as sulfate‐reducing bacteria are associated with hydrogen sulfide production, mucosal barrier disruption, and disease exacerbation. Thus, kefir intake may contribute to attenuating intestinal inflammation by selectively favoring beneficial microbial taxa while reducing potentially harmful ones.

Despite these positive outcomes, some limitations should be acknowledged. The duration of the intervention may not have been sufficient to induce detectable changes in cecal SCFA concentrations, suggesting that longer exposure or different dosing strategies could be necessary to fully assess functional microbial outputs. Moreover, although metataxonomic analysis revealed shifts in microbial composition, functional metagenomic or metabolomic approaches were not employed to directly link microbial changes with metabolic pathways. The use of a specific type of whole milk kefir also limits the generalizability of the findings.

Future studies should explore longer intervention periods, different kefir formulations, and dose–response relationships to better understand the functional impact of kefir on gut microbiota metabolism. Integrating multi‐omics approaches and evaluating additional inflammatory and immune markers may further clarify the mechanisms of the use of this probiotic with such potential protective effects. Ultimately, these findings contribute to the growing body of evidence supporting kefir as a promising functional food approach in the dietary management of intestinal inflammatory disorders.

## Author Contributions

**Iasmim Xisto Campos**: conceptualization, investigation, writing – original draft, methodology, validation, visualization, writing – review and editing, formal analysis, data curation, supervision. **Vinicius Fernandes Paris**: conceptualization, investigation, writing – original draft, methodology, validation, writing – review and editing, visualization, formal analysis, data curation. **Mariana de Fátima Albuquerque Pereira**: conceptualization, investigation, writing – original draft, methodology, validation, visualization, writing – review and editing, formal analysis, data curation. **Gabriela de Cássia Ávila Alpino**: conceptualization, investigation, writing – original draft, methodology, validation, visualization, writing – review and editing, formal analysis, data curation. **Andressa Ladeira Bernardes**: conceptualization, investigation, writing – original draft, methodology, validation, visualization, writing – review and editing, formal analysis, data curation. **Larissa Gabriela Morais de Ávila**: conceptualization, investigation, writing – original draft, methodology, validation, visualization, writing – review and editing, formal analysis, data curation. **Marcella Duarte Villas Mishima**: conceptualization, investigation, writing – original draft, methodology, validation, visualization, writing – review and editing, formal analysis, data curation. **Manoela Maciel dos Santos Dias**: conceptualization, writing – original draft, investigation, methodology, validation, visualization, writing – review and editing, formal analysis, data curation. **Soraya Torres Gaze Jangola**: conceptualization, investigation, writing – original draft, methodology, validation, visualization, writing – review and editing, supervision, resources, data curation, formal analysis. **Tiago Antonio de Oliveira Mendes**: conceptualization, investigation, writing – original draft, methodology, validation, visualization, writing – review and editing, formal analysis, supervision, resources, data curation. **Maria do Carmo Gouveia Peluzio**: conceptualization, investigation, writing – original draft, methodology, validation, visualization, writing – review and editing, formal analysis, project administration, data curation, supervision, resources.

## Conflicts of Interest

The authors declare no conflicts of interest.

## Data Availability

Illumina Hiseq raw sequences generated and analyzed during the current study are available in the Sequence Read Archive (SRA) database (https://www.ncbi.nlm.nih.gov/sra/PRJNA998822) under the BioProject PRJNA998822.

## References

[jfds70879-bib-0001] Ananthakrishnan, A. N. 2015. “Epidemiology and Risk Factors for IBD.” Nature Reviews Gastroenterology & Hepatology 12, no. 4: 205–217. https://www.nature.com/articles/nrgastro.2015.34. 10.1038/nrgastro.2015.34. 25732745

[jfds70879-bib-0002] Association of Official Analytical Chemistry . 2005. Official Methods of Analysis. Association of Official Analytical Chemistry.

[jfds70879-bib-0003] Azizi, N. , M. , Kumar , S. , Yeap , et al. 2021. “Kefir and Its Biological Activities.” Foods 10, no. 6: 1210. https://www.mdpi.com/2304‐8158/10/6/1210. 34071977 10.3390/foods10061210 PMC8226494

[jfds70879-bib-0004] Ministério Da Agricultura, Pecuária e Abastecimento . 2023. Instrução Normativa No 62, de 26 de Agosto de 2003. Oficializa Os Métodos Analíticos Oficiais Para Análises Microbiológicas Para Controle de Produtos de Origem Animal e Água. Ministério Da Agricultura, Pecuária e Abastecimento.

[jfds70879-bib-0005] Ministério Da Agricultura, Pecuária e Abastecimento . 2007. Resolução N° 46. Padrões de Identidade e Qualidade (PIQ) de Leites Fermentados. Ministério Da Agricultura, Pecuária e Abastecimento.

[jfds70879-bib-0006] Caporaso, J. , C. Lauber , W. A. Walters , et al. 2012. “Ultra‐High‐Throughput Microbial Community Analysis on the Illumina HiSeq and MiSeq Platforms.” ISME Journal 6, no. 8: 1621–1624. https://academic.oup.com/ismej/article‐abstract/6/8/1621/7587776. 22402401 10.1038/ismej.2012.8 PMC3400413

[jfds70879-bib-0007] Carson, F. , J. Martin , and J. A. Lynn . 1973. “Formalin Fixation for Electron Microscopy: A Re‐Evaluation.” American Journal of Clinical Pathology 59, no. 3: 365–373. https://academic.oup.com/ajcp/article‐abstract/59/3/365/1770672. 4119901 10.1093/ajcp/59.3.365

[jfds70879-bib-0008] Chen, H. , Y. Xia , S. Zhu , et al. 2017. “ *Lactobacillus plantarum* LP‐Onlly Alters the Gut Flora and Attenuates Colitis by Inducing Microbiome Alteration in Interleukin‐10 Knockout Mice.” Molecular Medicine Reports 16, no. 5: 5979–5985. 10.3892/MMR.2017.7351. 28849048 PMC5865777

[jfds70879-bib-0009] Chou, J. , H. Lai , C. Chang , K. S. Cheng , C. L. Feng , and T. W. Chen . 2019. “Epidemiology and Clinical Outcomes of Inflammatory Bowel Disease: A Hospital‐Based Study in Central Taiwan.” Gastroenterology Research and Practice 2019: 4175923. https://www.hindawi.com/journals/grp/2019/4175923/abs/. 31312216 10.1155/2019/4175923 PMC6595318

[jfds70879-bib-0010] Cruz, B. C. d. S. , L. L. d. Conceição , T. A. d. O. Mendes , C. L. d. L. F. Ferreira , R. V. Gonçalves , and M. d. C. G. Peluzio . 2020. “Use of the Synbiotic VSL#3 and Yacon‐Based Concentrate Attenuates Intestinal Damage and Reduces the Abundance of Candidatus Saccharimonas in a Colitis‐Associated Carcinogenesis Model.” Food Research International (Ottawa, Ontario) 137: 109721. https://pubmed.ncbi.nlm.nih.gov/33233290/. 10.1016/J.FOODRES.2020.109721. 33233290

[jfds70879-bib-0011] Dai, C. , Y. Huang , and M. Jiang . 2023. “Combination Therapy in Inflammatory Bowel Disease: Current Evidence and Perspectives.” International Immunopharmacology 114: 109545. https://www.sciencedirect.com/science/article/pii/S156757692201030X?casa_token=pzHxAJqYxgcAAAAA:7_BT1CSr5h‐Qb7ctN4IlnV6kGrs_Cq‐BuHGpXABTw5VZFE7b6Zr4_L2SbtdcHguiPCItQHA99Q. 36508920 10.1016/j.intimp.2022.109545

[jfds70879-bib-0012] Davidson, N. J. , M. W. Leach , M. M. Fort , et al. 1996. “T Helper Cell 1‐Type CD4+ T Cells, but Not B Cells, Mediate Colitis in Interleukin 10‐Deficient Mice.” Journal of Experimental Medicine 184, no. 1: 241–251. https://rupress.org/jem/article‐abstract/184/1/241/50734. 8691138 10.1084/jem.184.1.241 PMC2192682

[jfds70879-bib-0013] Dharmani, P. , V. Srivastava , V. Kissoon‐Singh , and K. Chadee . 2009. “Role of Intestinal Mucins in Innate Host Defense Mechanisms Against Pathogens.” Journal of Innate Immunity 1: 123–135. https://karger.com/jin/article‐abstract/1/2/123/180055. 10.1159/000163037. 20375571 PMC7312850

[jfds70879-bib-0014] Du, G. , S. Chang , Q. Guo , et al. 2022. “Protective Effects of Tibetan Kefir in Mice With Ochratoxin A‐Induced Cecal Injury.” Food Research International (Ottawa, Ontario) 158: 111551. https://pubmed.ncbi.nlm.nih.gov/35840245/. 10.1016/J.FOODRES.2022.111551. 35840245

[jfds70879-bib-0015] Farnworth, E. R. , ed. 2008. Handbook of fermented functional foods. CRC Press.

[jfds70879-bib-0016] Elson, C. O. , R. B. Sartor , G. S. Tennyson , and R. H. Riddell . 1995. “Experimental Models of Inflammatory Bowel Disease.” Gastroenterology 109, no. 4: 1344–1367. 10.1016/0016-5085(95)90599-5. 7557106

[jfds70879-bib-0017] Fu, Y. , J. Lyu , and S. Wang . 2023. “The Role of Intestinal Microbes on Intestinal Barrier Function and Host Immunity From a Metabolite Perspective.” Frontiers in Immunology 14: 1277102. 10.3389/FIMMU.2023.1277102. 37876938 PMC10591221

[jfds70879-bib-0018] Hadji, H. , and K. Bouchemal . 2022. “Advances in the Treatment of Inflammatory Bowel Disease: Focus on Polysaccharide Nanoparticulate Drug Delivery Systems.” Advanced Drug Delivery Reviews 181: 114101. https://www.sciencedirect.com/science/article/pii/S0169409x21004944?casa_token=MuOzt81oveMAAAAA:8lIty4g2PC4BelRJh41eCatqyM12KiorMtq2106Fe0joIFwNPs9l5tBZe6L6MXBcJGEStzHDLg. 34999122 10.1016/j.addr.2021.114101

[jfds70879-bib-0019] Haneishi, Y. , Y. Furuya , M. Hasegawa , A. Picarelli , M. Rossi , and J. Miyamoto . 2023. “Inflammatory Bowel Diseases and Gut Microbiota.” International Journal of Molecular Sciences 24, no. 4: 3817. https://www.mdpi.com/1422‐0067/24/4/3817. 36835245 10.3390/ijms24043817 PMC9958622

[jfds70879-bib-0020] Hee, B. v. d. , and J. M. Wells . 2021. “Microbial Regulation of Host Physiology by Short‐Chain Fatty Acids.” Trends in Microbiology 29, no. 8: 700–712. https://www.cell.com/trends/microbiology/fulltext/S0966‐842X(21)00035‐4. 33674141 10.1016/j.tim.2021.02.001

[jfds70879-bib-0021] Hou, T. , and E. Tako . 2018. “The In Ovo Feeding Administration (*Gallus gallus*)—An Emerging In Vivo Approach to Assess Bioactive Compounds With Potential Nutritional Benefits.” Nutrients 10, no. 4: 418. https://www.mdpi.com/2072‐6643/10/4/418. 29597266 10.3390/nu10040418 PMC5946203

[jfds70879-bib-0022] Instituto Adolfo Lutz . 1985. Métodos Químicos e Físicos Para Análise de Alimentos. Instituto Adolfo Lutz.

[jfds70879-bib-0023] Jandhyala, S. M. , R. Talukdar , C. Subramanyam , H. Vuyyuru , M. Sasikala , and D. N. Reddy . 2015. “Role of the Normal Gut Microbiota.” World Journal of Gastroenterology: WJG 21, no. 29: 8787. https://www.ncbi.nlm.nih.gov/pmc/articles/PMC4528021/. 26269668 10.3748/wjg.v21.i29.8787 PMC4528021

[jfds70879-bib-0024] Kim, Y. S. , and S. B. Ho . 2010. “Intestinal Goblet Cells and Mucins in Health and Disease: Recent Insights and Progress.” Current Gastroenterology Reports 12, no. 5: 319–330. 10.1007/S11894-010-0131-2. 20703838 PMC2933006

[jfds70879-bib-0025] Kostic, A. , R. Xavier , and D. Gevers . 2014. “The Microbiome in Inflammatory Bowel Disease: Current Status and the Future Ahead.” Gastroenterology 146, no. 6: 1489–1499. https://www.sciencedirect.com/science/article/pii/S0016508514002200?casa_token=rWv87CJ2tbcAAAAA:Z4ziOggCfecFbNCfEcZ6iXPOarJ_F7KN5Vyhx_SObkAMTH‐gzLpb_4syW877P6UJkk81CDCnlQ. 24560869 10.1053/j.gastro.2014.02.009 PMC4034132

[jfds70879-bib-0026] Kushkevych, I. , J. Cejnar , J. Treml , D. Dordević , P. Kollar , and M. Vítězová . 2020. “Recent Advances in Metabolic Pathways of Sulfate Reduction in Intestinal Bacteria.” Cells 9, no. 3: 698. https://www.mdpi.com/2073‐4409/9/3/698. 32178484 10.3390/cells9030698 PMC7140700

[jfds70879-bib-0027] Lê, A. , M. Mantel , J. Marchix , M. Bodinier , G. Jan , and M. Rolli‐Derkinderen . 2022. “Inflammatory Bowel Disease Therapeutic Strategies by Modulation of the Microbiota: How and When to Introduce Pre‐, Pro‐, Syn‐, or Postbiotics?” American Journal of Physiology. Gastrointestinal and Liver Physiology 323, no. 6: G523–G553. https://pubmed.ncbi.nlm.nih.gov/36165557/. 10.1152/AJPGI.00002.2022. 36165557

[jfds70879-bib-0028] Leite, A. , M. A. Miguel , R. S. Peixoto , A. S. Rosado , J. T. Silva , and V. M. Paschoalin . 2013. “Microbiological, Technological and Therapeutic Properties of Kefir: A Natural Probiotic Beverage.” Brazilian Journal of Microbiology 44, no. 2: 341–349. https://www.scielo.br/j/bjm/a/j7s8Vnc9qz6FkQKCjrNDGPb/?lang=en. 24294220 10.1590/S1517-83822013000200001 PMC3833126

[jfds70879-bib-0029] Liévin‐Le Moal, V. , and A. L. Servin . 2006. “The Front Line of Enteric Host Defense Against Unwelcome Intrusion of Harmful Microorganisms: Mucins, Antimicrobial Peptides, and Microbiota.” Clinical Microbiology Reviews 19, no. 2: 315–337. 10.1128/CMR.19.2.315-337.2006. 16614252 PMC1471992

[jfds70879-bib-0030] Lopitz‐Otsoa, F. , A. Rementeria , N. Elguezabal , and J. Garaizar . 2006. “Kefir: A Symbiotic Yeast‐Bacteria Community With Alleged Healthy Capabilities.” Revista Iberoamericana de Micologia 23, no. 2: 67–74. https://www.academia.edu/download/90303675/s1130‐1406_2806_2970016‐x20220826‐1‐1xwbduw.pdf, 2006. 16854180 10.1016/s1130-1406(06)70016-x

[jfds70879-bib-0031] Mera, R. , H. Thompson , and C. Prasad . 1998. “How to Calculate Sample Size for an Experiment: A Case‐Based Description.” Nutritional Neuroscience 1, no. 1: 87–91. 10.1080/1028415X.1998.11747217. 27405915

[jfds70879-bib-0032] Mishima, M. D. V. , B. P. d. Silva , M. J. C. Gomes , et al. 2023. “Effect of Chia Flour Associated With High Fat Diet on Intestinal Health in Female Ovariectomized Wistar Rats.” European Journal of Nutrition 62, no. 2: 905–919. 10.1007/S00394-022-03043-2. 36326862

[jfds70879-bib-0033] Mizoguchi, A. 2012. “Animal Models of Inflammatory Bowel Disease.” Progress in Molecular Biology and Translational Science 105: 263–320. 10.1016/B978-0-12-394596-9.00009-3. 22137435

[jfds70879-bib-0034] Mourad, F. H. , K. A. Barada , and N. E. Saade . 2017. “Impairment of Small Intestinal Function in Ulcerative Colitis: Role of Enteric Innervation.” Journal of Crohn's and Colitis 11, no. 3: 369–377. https://academic.oup.com/ecco‐jcc/article‐abstract/11/3/369/2333888. 10.1093/ecco-jcc/jjw162 27655154

[jfds70879-bib-0035] Naama, M. , S. Telpaz , A. Awad , et al. 2023. “Autophagy Controls Mucus Secretion From Intestinal Goblet Cells by Alleviating ER Stress.” Cell Host & Microbe 31: 433–446. https://www.cell.com/cell‐host‐microbe/pdf/S1931‐3128(23)00031‐8.pdf. 10.1016/j.chom.2023.01.006. 36738733 PMC10016318

[jfds70879-bib-0036] Nagao‐Kitamoto, H. , and N. Kamada . 2017. “Host‐Microbial Cross‐Talk in Inflammatory Bowel Disease.” Immune Network 17, no. 1: 1–12. https://www.ncbi.nlm.nih.gov/pmc/articles/PMC5334117/. 28261015 10.4110/in.2017.17.1.1 PMC5334117

[jfds70879-bib-0037] Ng, S. , H. Shi , N. Hamidi , et al. 2017. “Worldwide Incidence and Prevalence of Inflammatory Bowel Disease in the 21st Century: A Systematic Review of Population‐Based Studies.” Lancet 390, no. 10114: 2769–2778. https://www.thelancet.com/journals/lancet/article/PIIS0140‐6736(17)324480/fulltext. 29050646 10.1016/S0140-6736(17)32448-0

[jfds70879-bib-0038] Núñez‐Gómez, V. , R. González‐Barrio , and M. J. Periago . 2023. “Interaction Between Dietary Fibre and Bioactive Compounds in Plant By‐Products: Impact on Bioaccessibility and Bioavailability.” Antioxidants 12, no. 4: 9041–9053. https://www.mdpi.com/2076‐3921/12/4/976. 10.3390/antiox12040976 PMC10135553 37107351

[jfds70879-bib-0039] Otles, S. , and Ö. Çağındı . 2003. “Kefir: A Probiotic Dairy‐Composition, Nutritional and Therapeutic Aspects.” Pakistan Journal of Nutrition 2, no. 2: 54–59. http://translateyar.ir/wp‐content/uploads/2018/12/8721‐English.pdf,2003.

[jfds70879-bib-0040] Park, C. , S. B. Kim , S. H. Choi , and S. Kim . 2021. “Comparison of 16S RRNA Gene Based Microbial Profiling Using Five Next‐Generation Sequencers and Various Primers.” Frontiers in Microbiology 12: 715500. 10.3389/FMICB.2021.715500/FULL. 34721319 PMC8552068

[jfds70879-bib-0041] Pelaseyed, T. , J. H. Bergström , J. K. Gustafsson , et al. 2014. “The Mucus and Mucins of the Goblet Cells and Enterocytes Provide the First Defense Line of the Gastrointestinal Tract and Interact With the Immune System.” Immunological Reviews 260, no. 1: 8–20. 10.1111/IMR.12182. 24942678 PMC4281373

[jfds70879-bib-0042] Peluzio, M. , C. G. do , M. Dias , M. e. de , J. A. Martinez , and F. I. Milagro . 2021. “Kefir and Intestinal Microbiota Modulation: Implications in Human Health.” Frontiers in Nutrition 8: 638740. 10.3389/FNUT.2021.638740/FULL. 33693024 PMC7938729

[jfds70879-bib-0043] Pihurov, M. , B. Păcularu‐Burada , M. Cotârleţ , M. A. Vasile , and G. E. Bahrim . 2021. “Novel Insights for Metabiotics Production by Using Artisanal Probiotic Cultures.” Microorganisms 9, no. 11: 2184. https://www.mdpi.com/2076‐2607/9/11/2184. 34835310 10.3390/microorganisms9112184 PMC8624174

[jfds70879-bib-0044] Pittayanon, R. , J. Lau , G. Leontiadis , et al. 2020. “Differences in Gut Microbiota in Patients With vs Without Inflammatory Bowel Diseases: A Systematic Review.” Gastroenterology 158, no. 4: 930–946. https://www.sciencedirect.com/science/article/pii/S0016508519418933?casa_token=K1JjnEmZlckAAAAA:7ymrs7BLWzpkkTEyqRGNtK5q47iKztrkSnB5u6S_TPgh6ujOCiBwAU3Iwf67INd8PFJBw‐fFMQ,2020. 31812509 10.1053/j.gastro.2019.11.294

[jfds70879-bib-0045] van der Post, S. , K. S. Jabbar , g. Birchenough , et al. 2019. “Structural Weakening of the Colonic Mucus Barrier Is an Early Event in Ulcerative Colitis Pathogenesis.” Gut 68, no. 12: 2142–2151. https://gut.bmj.com/content/68/12/2142.abstract. 10.1136/gutjnl-2018-317571. 30914450 PMC6872445

[jfds70879-bib-0046] Prado, M. R. , L. M. Blandón , L. P. S. Vandenberghe , et al. 2015. “Milk Kefir: Composition, Microbial Cultures, Biological Activities, and Related Products.” Frontiers in Microbiology 6: 1177. 10.3389/FMICB.2015.01177/FULL. 26579086 PMC4626640

[jfds70879-bib-0047] Qiu, X. , M. Macchietto , X. Liu , et al. 2021. “Identification of Gut Microbiota and Microbial Metabolites Regulated by an Antimicrobial Peptide Lipocalin 2 in High Fat Diet‐Induced Obesity.” International Journal of Obesity 45, no. 1: 143–154. https://www.nature.com/articles/s41366‐020‐00712‐2. 33214705 10.1038/s41366-020-00712-2 PMC7755824

[jfds70879-bib-0048] Qu, Y. , X. Li , F. Xu , et al. 2021. “Kaempferol Alleviates Murine Experimental Colitis by Restoring Gut Microbiota and Inhibiting the LPS‐TLR4‐NF‐ΚB Axis.” Frontiers in Immunology 12: 679897. 10.3389/FIMMU.2021.679897/FULL. 34367139 PMC8339999

[jfds70879-bib-0049] Quast, C. , E. Pruesse , P. Yilmaz , et al., 2013. “The SILVA Ribosomal RNA Gene Database Project: Improved Data Processing and Web‐Based Tools.” Nucleic Acids Research 41, no. D1: D590–D596. https://academic.oup.com/nar/article‐abstract/41/D1/D590/1069277. 23193283 10.1093/nar/gks1219 PMC3531112

[jfds70879-bib-0050] Rosa, D. D. , M. M. S. Dias , Ł. M. Grześkowiak , et al. 2017. “Milk Kefir: Nutritional, Microbiological and Health Benefits.” Nutrition Research Reviews 30: 82–96. https://www.cambridge.org/core/journals/nutrition‐research‐reviews/article/milk‐kefir‐nutritional‐microbiologic‐and‐health‐benefits/1393DC2B8E5F08B0BE7BD58F030D387B. 10.1017/S0954422416000275. 28222814

[jfds70879-bib-0051] Rosa, D. D. , L. M. Grześkowiak , C. L. L. F. Ferreira , et al. 2016. “Kefir Reduces Insulin Resistance and Inflammatory Cytokine Expression in an Animal Model of Metabolic Syndrome.” Food & Function 7, no. 8: 3390–3401. https://pubs.rsc.org/en/content/articlehtml/2016/fo/c6fo00339g. 10.1039/C6FO00339G. 27384318

[jfds70879-bib-0052] Rosa, D. D. , F. C. Loureno , A. C. M. H. D. Fonseca , et al. 2012. “Fish Oil Improves the Lipid Profile and Reduces Inflammatory Cytokines in Wistar Rats With Precancerous Colon Lesions.” Nutrition and Cancer 64, no. 4: 569–579. 10.1080/01635581.2012.665563. 22483364

[jfds70879-bib-0053] Russo, E. , F. Giudici , C. Fiorindi , F. Ficari , S. Scaringi , and A. Amedei . 2019. “Immunomodulating Activity and Therapeutic Effects of Short Chain Fatty Acids and Tryptophan Post‐Biotics in Inflammatory Bowel Disease.” Frontiers in Immunology 10: 2754. 10.3389/FIMMU.2019.02754/FULL. 31824517 PMC6883404

[jfds70879-bib-0054] Sairenji, T. , K. Collins , and D. V. Evans . 2017. “An Update on Inflammatory Bowel Disease.” Primary Care: Clinics in Office Practice 44, no. 4: 673–692. https://www.primarycare.theclinics.com/article/S0095‐4543(17)30104‐5/abstract,2017. 29132528 10.1016/j.pop.2017.07.010

[jfds70879-bib-0055] Sarkar, S. 2008. “Biotechnological Innovations in Kefir Production: A Review.” British Food Journal 110, no. 3: 283–295. 10.1108/00070700810858691/FULL/HTML.

[jfds70879-bib-0056] Selvamani, S. , V. Mehta , H. Ali El Enshasy , et al. 2022. “Efficacy of Probiotics‐Based Interventions as Therapy for Inflammatory Bowel Disease: a Recent Update.” Journal of Biological Sciences 29, no. 5: 3546–3567. https://www.sciencedirect.com/science/article/pii/S1319562x22001206. 10.1016/j.sjbs.2022.02.044 PMC9280206 35844369

[jfds70879-bib-0057] Siddiqui, M. T. , and G. A. M. Cresci . 2021. “The Immunomodulatory Functions of Butyrate.” Journal of Inflammation Research 14: 6025–6041. https://pmc.ncbi.nlm.nih.gov/articles/PMC8608412/. 10.2147/JIR.S300989. 34819742 PMC8608412

[jfds70879-bib-0058] Siegfried, V. , H. Ruckermann , G. Stumpf , et al. 1984. “Method for the Determination of Organic Acids in Silage by High Performance Liquid Chromatography.” Landwirtsch Forsch 37, no. 3‐4: 298–304.

[jfds70879-bib-0059] Singh, S. B. , C. N. Coffman , M. G. Varga , A. Carroll‐Portillo , C. A. Braun , and H. C. Lin . 2022. “Intestinal Alkaline Phosphatase Prevents Sulfate Reducing Bacteria‐Induced Increased Tight Junction Permeability by Inhibiting Snail Pathway.” Frontiers in Cellular and Infection Microbiology 12: 00. 10.3389/FCIMB.2022.882498/FULL. PMC9177943 35694541

[jfds70879-bib-0060] Slattery, C. , P. D. Cotter , and P. W. O'Toole . 2019. “Analysis of Health Benefits Conferred by Lactobacillus Species From Kefir.” Nutrients 11, no. 6: 1252. 10.3390/NU11061252. 31159409 PMC6627492

[jfds70879-bib-0061] de Souza, H. F. , G. F. Monteiro , L. T. Bogáz , E. N. S. Freire , K. N. Pereira , et al. 2024. “Bibliometric Analysis of Water Kefir and Milk Kefir in Probiotic Foods From 2013 to 2022: A Critical Review of Recent Applications and Prospects.” Food Research International 175: 113716. 10.1016/J.FOODRES.2023.113716. 38128984

[jfds70879-bib-0062] Stojanov, S. , A. Berlec , and B. Štrukelj . 2020. “The Influence of Probiotics on the Firmicutes/Bacteroidetes Ratio in the Treatment of Obesity and Inflammatory Bowel Disease.” Microorganisms 8, no. 11: 1715. https://www.mdpi.com/2076‐2607/8/11/1715. 33139627 10.3390/microorganisms8111715 PMC7692443

[jfds70879-bib-0063] Venegas, D. P. , L. Fuente , M. K. De , et al. 2019. “Short Chain Fatty Acids (SCFAs)‐Mediated Gut Epithelial and Immune Regulation and Its Relevance for Inflammatory Bowel Diseases.” Frontiers in Immunology 10: 277. 10.3389/FIMMU.2019.00277/FULL. 30915065 PMC6421268

[jfds70879-bib-0064] Vujičić, I. F. , M. Vulić , and T. Könyves . 1992. “Assimilation of Cholesterol in Milk by Kefir Cultures.” Biotechnology Letters 14, no. 9: 847–850. 10.1007/BF01029151.

[jfds70879-bib-0065] Xue, J. , J. A. Dominguez Rieg , L. Thomas , J. R. White , and T. Rieg . 2022. “Intestine‐Specific NHE3 Deletion in Adulthood Causes Microbial Dysbiosis.” Frontiers in Cellular and Infection Microbiology 12: 896309. 10.3389/FCIMB.2022.896309/FULL. 35719363 PMC9204535

[jfds70879-bib-0066] Zhai, Z. , F. Zhang , R. Cao , et al. 2019. “Cecropin A Alleviates Inflammation Through Modulating the Gut Microbiota of C57BL/6 Mice With DSS‐Induced IBD.” Frontiers in Microbiology 10: 1595. 10.3389/FMICB.2019.01595. 31354682 PMC6635700

[jfds70879-bib-0067] Zhang, B. W. , M. Li , L. C. Ma , and F. W. Wei . 2006. “A Widely Applicable Protocol for DNA Isolation From Fecal Samples.” Biochemical Genetics 44, no. 11–12: 494–503. 10.1007/S10528-006-9050-1. 17094033

